# Extracellular Vesicles and Thrombosis: Update on the Clinical and Experimental Evidence

**DOI:** 10.3390/ijms22179317

**Published:** 2021-08-27

**Authors:** Konstantinos Zifkos, Christophe Dubois, Katrin Schäfer

**Affiliations:** 1 Center for Thrombosis and Hemostasis, University Medical Center Mainz, D-55131 Mainz, Germany; k.zifkos@uni-mainz.de; 2 Aix Marseille University, INSERM 1263, Institut National de la Recherche pour l’Agriculture, l’alimentation et l’Environnement (INRAE) 1260, Center for CardioVascular and Nutrition Research (C2VN), F-13380 Marseille, France; christophe.dubois@univ-amu.fr; 3 Department of Cardiology, Cardiology I, University Medical Center Mainz, D-55131 Mainz, Germany

**Keywords:** arterial, endothelial cells, extracellular vesicles, thrombosis, venous

## Abstract

Extracellular vesicles (EVs) compose a heterogenous group of membrane-derived particles, including exosomes, microvesicles and apoptotic bodies, which are released into the extracellular environment in response to proinflammatory or proapoptotic stimuli. From earlier studies suggesting that EV shedding constitutes a cellular clearance mechanism, it has become evident that EV formation, secretion and uptake represent important mechanisms of intercellular communication and exchange of a wide variety of molecules, with relevance in both physiological and pathological situations. The putative role of EVs in hemostasis and thrombosis is supported by clinical and experimental studies unraveling how these cell-derived structures affect clot formation (and resolution). From those studies, it has become clear that the prothrombotic effects of EVs are not restricted to the exposure of tissue factor (TF) and phosphatidylserines (PS), but also involve multiplication of procoagulant surfaces, cross-linking of different cellular players at the site of injury and transfer of activation signals to other cell types. Here, we summarize the existing and novel clinical and experimental evidence on the role and function of EVs during arterial and venous thrombus formation and how they may be used as biomarkers as well as therapeutic vectors.

## 1. Introduction

Extracellular vesicles (EVs) are lipid bilayer structures that compose a heterogenous group of membrane-derived particles, including exosomes, microvesicles and apoptotic bodies, which are released into the extracellular environment in response to cell activation or apoptosis. The diversity of EVs lies in differences in the mechanisms of their biogenesis as well as their cellular origin. EVs have been identified as mediators of cell-to-cell communication due to their ability to contain and transfer bioactive molecular cargo, such as transmembrane and cytosolic proteins, coding and non-coding RNA, or DNA, to both distant and neighboring cells. Their capacity of exchanging components between cells and their presence in the majority of body fluids suggests that they participate in normal homeostatic processes as well as pathological disease states, but also that they may serve as biomarkers and therapeutic vehicles. In addition, EVs may communicate with cells and affect their phenotype or function via surface molecule-triggered uptake and intracellular signaling. In the cardiovascular system, the release of EVs from activated endothelial cells, platelets and neutrophils, but also from monocytes and erythrocytes may transfer prothrombotic ‘message’ and promote blood clot formation, both in the arterial and the venous system.

In this review, we summarize the existing and novel clinical and experimental evidence on the role and function of EVs during arterial and venous thrombus formation and how they may be used as biomarkers as well as therapeutic vectors.

## 2. Extracellular Vesicles: Definition and Characterization

In general, all cell types are able to form and release various forms of EVs, that is submicron membrane fragments, into the extracellular space. Insight on the biogenesis of EVs was provided by means of transmission and immuno-electron microscopy. Based on these initial experiments and current knowledge, EVs are classified into exosomes, microvesicles and apoptotic bodies, taking into account their size, biogenesis pathway and localization outside the cell.

The term **‘exosome’** was first proposed in 1981 to refer to membrane vesicles with an average diameter of 500–1000 nm being ‘exfoliated’ from both normal and neoplastic cell lines and carrying ecto-5′-nucleotidase activity as well as high amounts of sphingomyelin [1]. Later, the term was used for 50 nm sized membrane vesicles expressing the transferrin receptor that were released from reticulocytes during maturation [2]. With a size ranging from 50 to 150 nm in diameter, they constitute the smallest group of EVs. Exosomes are formed by the inward budding of the membrane of intracellular (multivesicular) endosomes, which are released into the extracellular space after fusion with the outer cell membrane [3]. Exosomes are abundantly secreted by a variety of different cell types under both physiological and pathological conditions and can be detected in blood and many other body fluids. Since their discovery, the general perception of exosomes being just a means of clearing unwanted, obsolete intracellular material [4] has been expanded by the exosomal involvement in intercellular communication and the control of diverse biological pathways [5].

The second class of EVs, termed **‘microvesicles’** (MVs), also known as **microparticles** (MPs), shedding microvesicles or ectosomes, was initially described as ‘dust’ originating from platelets [6] and studied for its role in blood coagulation [7]. MVs range in size from 50 to 1000 nm in diameter. In contrast to exosomes, MVs are generated by the outward budding and shedding of the plasma membrane. This process does not only occur in platelets but can be observed in many other cell types. Originally, it was thought that MVs, like exosomes, represent a cellular homeostasis maintenance mechanism to get rid of unnecessary material. However, two studies played pivotal roles in discriminating the biological function of MVs from being just a dumping machinery to an intercellular communication mechanism. Initially, Giesen et al. demonstrated that tissue factor (TF), the main initiator of the extrinsic coagulation cascade, is released from neutrophils and monocytes in circulating blood and can be found in large clusters of microvesicles near the surface of platelets [8]. The ability of MVs to reprogram the phenotype of the recipient cell was established by another study showing that MVs from murine embryonic stem cells promote the survival of adult hematopoietic progenitor cells by the transfer of pluripotency transcription factors and induction of p42/44 mitogen-activated protein kinase (MAPK) and protein kinase B signaling [9]. Due to their biogenesis via cell membrane blebbing, the expression of specific membrane antigens that reflect their cellular origin is an important feature of this class of extracellular vesicles, a feature exploited using flow cytometry detection of cell-specific surface antigens. MVs are present in the blood of healthy individuals, but their numbers and cellular sources are altered in various pathological states, including cardiovascular disease, as outlined in more detail below.

A particular group of EVs are so-called **‘apoptotic bodies’**, which are generated by the vesiculation of apoptotic or dying cells [10]. With sizes ranging from 50 to 5000 nm, apoptotic bodies constitute the largest class of EVs. They are formed and released as result of cellular contractions and the increased hydrostatic pressure present in dying cells which lead to separation of the plasma membrane from the cytoskeleton [11]. Apoptotic bodies are cleared by phagocytosis, a process that facilitates the horizontal transfer of DNA fragments, including those that contain oncogenes [12]. In addition to cells of the innate immune system, vascular cells may also phagocytose extracellular material. For example, apoptotic bodies derived from dying endothelial cells have been shown to be taken up by the surviving endothelium via annexin I and to promote target cell survival through inhibition of p38 MAPK activity [13].

Since EVs are typically isolated by differential centrifugation and separated from other non-EV components based on their density and size, they may be further distinguished into small EVs (centrifugation speed: 100,000–200,000 g), which includes exosomes, and large EVs (centrifugation speed: 10,000–20,000 g), which includes MVs [14]. Although the majority (>95%) of EVs are spherical (30 nm to 1 µm in diameter), subfractions of EVs in platelet-free plasma preparations were found to exhibit a tubular shape (<5%; 1–5 µm long) or to resemble membrane fragments (<0.05%; 1–8 µm large) [15]. Being of similar size as anuclear platelets (2–3 µm in diameter) or erythrocytes (6–8 µm in diameter), small EVs may be difficult to distinguish from blood cells (or their fragments), if only size is considered as the main distinguishing feature.

For more details on the standards in the isolation and characterization of EVs, the reader is referred to comprehensive reviews or position statements on this topic [5,16]. Please note that we decided to keep the name chosen by the authors to describe the type of EVs examined in their work and did not change it to the generic term ‘extracellular vesicle’, introduced by the International Society for Extracellular Vesicles (ISEV) as an overarching description of all types of membrane vesicles [16,17]. The importance of methodological standardization and technical developments over time is highlighted by the observation of procoagulant properties of EVs from healthy individuals in 2001 [18], whereas the re-evaluation of their functional properties by the same group in 2019 revealed that EVs in blood of healthy humans promote fibrinolysis rather than coagulation [19].

## 3. Mechanisms of EV Formation

### 3.1. General

In healthy individuals, the concentrations of total EVs in plasma have been reported to be in the range of 10^3^–10^11^ per mL [20,21] and thus to vary widely, possibly depending on the methodology used for their quantification (flow cytometry, nanoparticle tracking, other) and its respective sensitivity. Increased EV or MP levels are observed in disease states associated with a prothrombotic tendency, such as thrombotic thrombocytopenic purpura [22], heparin-induced thrombocytopenia [23] or sickle cell disease [24], but also in patients with cardiovascular risk factors, such as diabetes mellitus [25] and metabolic syndrome [26], or established cardiovascular disease, such as acute coronary syndrome (ACS) [27] and stroke [28]. Whether increased circulating EV numbers are ‘just another biomarker’ of activation or injury of the cell of origin, and possibly also of an increased risk of thrombosis in the future, or whether they actively participate in disease processes is not entirely clear and may be dependent on the type of pathology. For example, EVs are also elevated in patients with cancer and have been shown to play a role in tumor progression and metastasis [29,30].

EVs, and in particular MPs, are generated by plasma membrane blebbing, a process controlled by the lipid composition of the cell membrane, among others and also the functional organization of the cytoskeleton. For that reason, MPs display abnormal lipid arrangements, such as the externalization of phosphatidylserine (PS), a phospholipid component of the cell membrane localized exclusively in the cytoplasmic leaflet where it forms part of protein docking sites necessary for the activation of important cell signaling pathways [31]. During cell activation or death, PS are actively translocated to the exoplasmic leaflet and exposed on the cell surface to initiate apoptotic cell clearance [32]. The process of PS exposure is triggered by bivalent cations and may be induced experimentally by the Ca^2+^ ionophore A23187 (also known as calcimycin) or 2,5-di-(t-butyl)-1, 4-benzohydroquinone, a Ca^2+^-ATPase inhibitor. Of note, about 50% of EVs expressing platelet (i.e., CD41) or erythrocyte (i.e., CD235a) markers were found not to expose PS, as indirectly determined by quantification of annexin V binding [33], suggesting that they are generated via mechanisms in which the plasma membrane lipid asymmetry is maintained [15]. An alternative explanation is that PS exposure did not reach the threshold required for measurable annexin V binding [34].

Numerous studies in vitro and in vivo reported on the impact of certain stimuli on the release of EVs, thereby not only providing insights into their contribution to the onset and progression of disease, but also shedding light on potential therapeutic options involving the modulation of EV release or their cargo. A summary of commonly used experimental stimuli to induce the release of EVs from vascular cells is given in Table 1. Notably, different cell types treated by the same stimulus may release distinct EVs, and the type of stimulus also determines their effects on the recipient cell, as outlined in more detail in the next section.

### 3.2. Activation and Inflammation

Inflammatory cytokines promote the formation of EVs from various cell types, including endothelial cells and monocytes. Most prominent among those is the proinflammatory cytokine tumor necrosis factor-alpha (TNFα), used in many studies as a model agent to study EV formation [39,40,57]. Other examples of proinflammatory stimuli include lipopolysaccharide (LPS), but also reactive oxygen species (ROS), oxidized low density lipoproteins or angiotensin II. Activation of the MAPK signaling pathway, in particular its component p38 MAPK, has been implicated in the generation and release of endothelial EVs in response to proinflammatory stimuli, such as TNFα [38]. Whereas some studies found that p38 inhibition (using SB-203580) prevented the upregulation of adhesion proteins and monocyte adherence on human coronary artery endothelial cells elicited by endothelial MPs exposed to high glucose [58], others found that p38 MAPK inhibition (using SB-239063) reduced MP release from human aortic endothelial cells (HAECs) in response to TNFα, but did not prevent their inflammatory response [38]. A reduction in the release of inflammatory MPs from TNFα-stimulated endothelial cells could also be achieved by silencing of TNF receptor-1 or by inhibiting nuclear factor kB [57]. P38 MAPK as well as c-Jun N-terminal kinase-1 have also been implicated in endothelial MP generation elicited by thrombin and CD40L [47], underlining the close interaction between inflammation and thrombosis. Established mediators of activation-induced cell death, that is Fas (CD95) ligand (FasL) and TRAIL (APO2 ligand/TNF-related apoptosis-inducing ligand), are involved in the release of procoagulant MPs from endothelial cells in response to thrombin [48]. Interestingly, sex differences in the expression of cell adhesion molecules on MVs released from human brain microvascular endothelial cells in response to TNFα and thrombin have been observed [42], although the underlying mechanisms and relevance for endothelium-mediated pathologies in humans have yet to be determined.

The concept that elevated circulating endothelial EVs are not only useful as a biomarker of endothelial injury, but actively participate in the progression of cardiovascular disease, is supported by findings that MPs derived from activated endothelial cells may transfer dysfunction to untreated endothelial recipient cells [58], for example by reducing the bioavailability of nitric oxide (NO) [59] or impairing endothelial-dependent vasorelaxation [60].

### 3.3. Age and Senescence

Cellular senescence is associated with an enhanced biogenesis and release of EVs [61], as first shown in normal human prostate cancer cells following induction of senescence by telomere attrition (e.g., aging) or DNA damage (e.g., irradiation) [62]. Other cellular stressors, such as heat shock, hypoxia, hypothermia or oxidative stress, also have been reported to increase EV secretion and to induce changes in EV composition [63]. Findings that inhibition of exosome biosynthesis and release from senescent cells promotes DNA damage and accelerates apoptosis-like death [64] suggest that the EV-mediated release of harmful DNA fragments and other factors during cellular senescence may be protective and prevent innate immune responses. The ability of EVs to transfer cell-specific cargo to bystander, recipient cells and to influence their phenotype and function has already been mentioned in one of the previous sections. In this context, EVs produced and secreted from senescent cells have their own unique characteristics and may be part of the senescent-associated secretory phenotype [65].

Whether these observations in senescent cells translate into aging humans, is another question. Eitan et al. used nanoparticle tracking analysis (NTA) to measure EV numbers in 30 young, 30 middle-aged and 14 older people and found that EV numbers in plasma decreased with advancing age [66]. The authors speculate that the decreased concentration of EVs with age may be due, at least in part, to their increased internalization by leukocytes. Other potential explanations include the slow turnover of senescent cells. Forest et al. also observed decreased levels of circulating endothelial MPs in elderly people compared with healthy young individuals. However, endothelial EVs from the elderly maintained their procoagulant activity contrary to that of young control subjects [67]. Further studies are necessary to clarify the relevance of this in vitro observation for the increased risk of thrombosis associated with age.

### 3.4. Cell Death and Apoptosis

During apoptotic death, cells secrete so-called apoptotic cell-derived EVs (Apo-EVs) that act as carriers of nucleic acids, proteins and lipids contained in dying cells and actively transmit them to neighboring or distant cells by entering the circulation [68]. Contrary to other forms of cellular EVs, Apo-EVs mediate intercellular communication by enveloping the remaining components of dead cells, including nucleic acids, proteins and lipids, and transferring them to recipient cells [69]. The effects of this process range from tissue regeneration to the horizontal transfer of DNA and subsequent changes in the cellular phenotype. For example, it was shown that DNA cargo contained in endothelial cell-derived Apo-EVs enhance the proliferation and differentiation of human endothelial progenitor cells [69]. In the same manner, it was observed that amino acids enveloped by Apo-EVs and released from fibroblasts were able to induce gene recombination [70] or that microRNA (miR) contained in macrophage-derived Apo-EVs promoted the proliferation of lung epithelial cells [71]. So far, a direct role for Apo-EVs during thrombus formation has not been reported.

## 4. Fate and Effects of EVs on Recipient Cells

EVs carry molecular bioactive cargo and are capable of changing the phenotype and function of the recipient cells. In order to exert these effects, EVs have to be deposited on the recipient cells to induce intracellular signaling events or to be taken up, either by direct fusion with the plasma membrane or by endocytosis and fusion with membranes of the endosomal compartment. The mechanisms involved have been reviewed in detail elsewhere [72,73] and are therefore only briefly discussed here.

The interaction of EVs with the recipient cell involves their adhesion and binding to molecules or receptors expressed on the surface of target cells. One group of proteins that claim a particular role in the cellular uptake of EVs are tetraspanins. Tetraspanins are membrane proteins involved in cell adhesion, activation and proliferation [74] and abundantly expressed on the surface of EVs. In fact, members of this group of proteins (e.g., CD9, CD63, CD81) are recommended by the ISEV as general EV markers [16]. In addition, tetraspanins may recruit other adhesion receptors, such as integrins, and thus organize and fine-tune the interaction of EVs with counter receptors expressed on their targets, as shown for CD81, integrin α4β1 and VCAM-1 on endothelial cells [75], for CD63 as co-factor necessary for the release of P-selectin from Weibel-Palade bodies [76] and for CD9 for the regulation of their adhesive properties and interaction with integrin αLβ2 on leucocytes [77]. Integrin receptors play also pivotal roles in cell–extracellular matrix and cell–cell interactions during blood clot formation. Abciximab, the c7E3 Fab fragment directed against integrin αIIbβ3 routinely given in patients to prevent platelet aggregation and thrombotic complications following vascular injury, was shown to inhibit the uptake of PS-positive MPs by human endothelial cells [78]. Immunoglobulins, such as intracellular adhesion molecule-1 (ICAM-1), proteoglycans, such as heparan sulfate proteoglan receptors, glycoproteins, such as the hyaluron receptor CD44, or lectins constitute other groups of proteins that support the docking and uptake of EVs by recipient cells [73]. The composition and expression of ligands and counter receptors on EVs may direct and determine the specificity of their cellular interactions and explain the recognition and preferential uptake of EVs by certain cell types and organ systems [79]. Of note, changes in circulating EV numbers may not only reflect increased production, but also indicate defective clearance mechanisms. In this regard, defects in PS-positive MP clearance from the circulation, as observed in lactadherin-deficient mice, were found to be associated with a hypercoagulable state [80].

Although EVs may stay bound on the cell surface and induce cellular signaling events without being internalized, evidence from the majority of studies indicates that EVs and particularly exosomes, are rapidly taken up by recipient cells, either by direct fusion with the plasma membrane or via endocytosis, and act by delivering their cargo to the recipient cell. It has been shown that EVs can be identified intracellularly as early as 15 min after their introduction to the target cells [81]. The majority of studies suggested that the swift transition of EVs from the extracellular environment towards their cellular internalization is dependent on an active, energy-consuming process. Findings from different groups have shown that the capacity to internalize EVs is dramatically reduced when cells were incubated either at 4 [3] or 48 °C [73]. Heat treatment at 65 °C for 60 min completely abolished the procoagulant activity of endothelial MPs [82]. Formalin fixation, treatment with the actin-disrupting agents cytochalasin D or latrunculin B or with calcium chelators also halt the cellular uptake process of EVs [73].

It should be noted that EV uptake may occur through more than one mechanism and that this is also dependent on their respective subtype. In this regard, clathrin-independent endocytosis and macro-pinocytosis [83] or phagocytosis [84] all have given evidence of potential participation in their internalization process. In endothelial cells, chlorpromazine, an inhibitor of clathrin-dependent endocytosis, was shown to reduce the uptake of PS-positive microparticles (MPs) to 20%, whereas amiloride, an inhibitor of micropinocytosis, showed no effect [78]. Additionally, non-classical endocytosis of EVs via intact lipid rafts and caveolea has been reported. For example, cholesterol depletion [85], pharmacological inhibition of caveolae endocytosis [85] or knockdown of caveolin-1 [83,86], a major constituent of lipid rafts, all have been shown to reduce MP and exosome uptake by target cells.

The involvement of EVs in pathological conditions and their emerging therapeutic potential as drug delivery vehicle has underlined the importance of understanding the molecular mechanisms that regulate their cellular uptake. Using fluorescently labeled exosomes it was shown that they are taken up by virtually every cell type tested [86], whereas other studies indicated that the internalization process is only possible if EVs and the recipient cell share the right combination of receptor and ligand [73]. The heterogeneity of these observations suggests that the types of EVs and recipient cells, the experimental setup and the individual purpose of each experiment, all are important factors that influence processes involved in cellular internalization of EVs.

## 5. Extracellular Vesicles and Thrombosis

**Figure 1:**
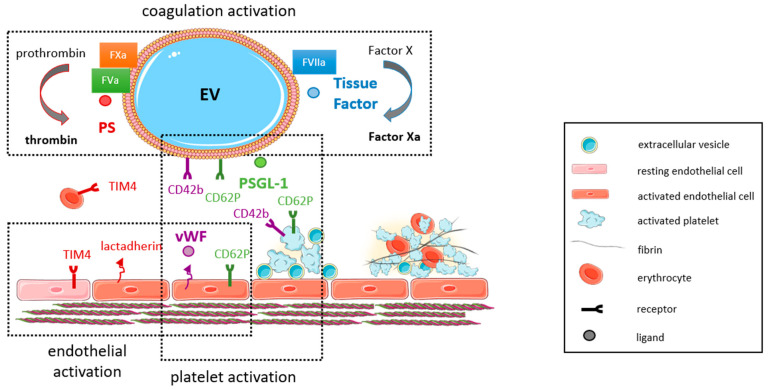
**Schematic drawing depicting the main pathways and mediators of thrombus formation affected by extracellular vesicles**. Extracellular vesicles (EVs) are shed from activated or apoptotic cells and may promote activation of the coagulation cascade by exposing phosphatidylserines (PS) and formation of the prothrombinase complex (together with factors Va and Xa) resulting in thrombin activation and by the release of tissue factor and activation of factor X (together with factor VII). Endothelial cells and erythrocytes express the PS receptor TIM4 as well as the receptor for PSGL-1 (CD62P), which also is upregulated on activated platelets. EVs and platelets also express counter receptors (e.g., CD42b) for coagulation proteins (e.g., von Willebrand factor, vWF). The reciprocal interaction and cross-talk between EVs and vascular cells, platelets and coagulation proteins ultimately results in blood clot formation. This figure was generated with the help of https://smart.servier.com/ (accessed on 25 July 2021).

EVs, being released from activated, senescent or apoptotic cells, are deeply involved in the pathomechanisms of thrombosis, both in the arterial and the venous system (Figure 1). Not only are they released in response to known prothrombotic stimuli, but they may themselves exert prothrombotic activities. During thrombosis, vascular and blood cells interact directly, but they also communicate and connect through EVs released from the different cell types involved in blood clot formation. Ex vivo studies performed in the Badimon perfusion chamber revealed that the enrichment of human blood with circulating MPs isolated from healthy subjects significantly increased the adhesion of platelets and deposition of fibrin on damaged porcine aorta and human atherosclerotic arteries [87].

The prothrombotic characteristics of MPs mainly rely on the expression of procoagulant proteins, such as TF [8], and the exposure of negatively charged phospholipids, such as PS [88], on their surfaces. PS-exposing EVs are considered procoagulant, because PS facilitates the assembly of tenase (factors VIIIa, IXa, X) and prothrombinase (factors Va, Xa, thrombin) complexes in the presence of calcium ions [89]. In addition, PS-positive, apoptotic EVs may directly associate with factor XII and support thrombin generation via activation of the intrinsic coagulation cascade [90]. TF and its inhibitor, tissue factor pathway inhibitor (TFPI), are stored and concentrated in caveolae [91,92], from where they are released together with MPs [84]. Exposure of TF was observed on MPs from platelets, erythrocytes, neutrophil granulocytes and monocytes, and preincubation with a TF-neutralizing antibody abolished their ability to promote fibrin generation in vitro [93]. Other procoagulant molecules and receptors carried by MPs and enabling thrombus propagation include P-selectin glycoprotein ligand-1 (PSGL-1, CD162), which also is present in lipid rafts [84] and mediates the interaction of MPs with CD62P expressed on activated platelets and endothelial cells [94]. Von Willebrand factor (vWF), released from Weibel-Palade bodies of injured or activated endothelial cells, recruits procoagulant platelet EVs by binding to glycoprotein GpIb [95], a mechanism involved in coagulopathy associated with traumatic brain injury [96]. Of note, thrombin-generating cell-derived MPs also circulate in blood of healthy humans, although their effects on coagulation were found to occur via TF-independent pathways and were partially inhibited using antibodies against factor XII, XI or VIII [18].

The pattern of surface procoagulant factor expression strongly depends on the cellular origin, the type of stimulus and the mechanism of EV formation. In the context of their contribution to thrombosis, we will focus on EVs released from cell types known to be involved in blood clot formation and resolution and therefore briefly introduce EVs released from endothelial cells, platelets, erythrocytes and cells of the innate immune system (monocytes, granulocytes). Insights into the ability of EVs to activate coagulation, their relative contributions in this process and the importance of their cellular origin have come from ex vivo studies using the Thrombodynamics test assay, and the potential to activation coagulation was found to be highest in monocyte-derived MPs, followed by MPs released from endothelial cells, platelets and erythrocytes [97], and similar findings as well as the role of active TF in this observations was demonstrated by others [98].

**Figure 2:**
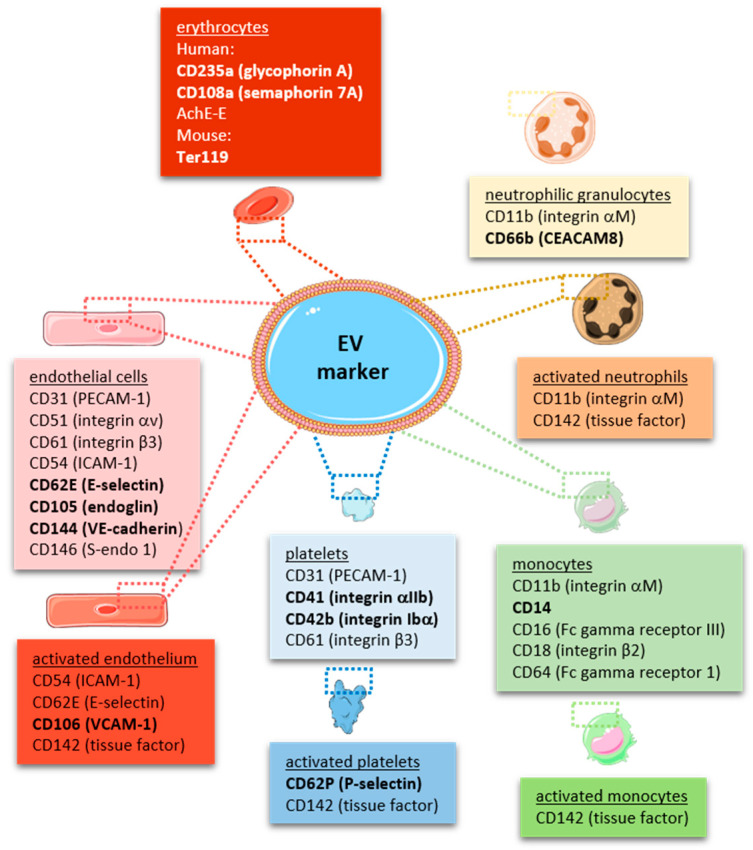
**Markers of extracellular vesicles originating from cells involved in thrombosis and hemostasis.** Schematic drawing summarizing the most common markers used to characterize the cellular origin of extracellular vesicles (EVs), focusing on cells involved in coagulation and bleeding control. The most specific markers are highlighted in ‘bold’. This figure was generated with the help of https://smart.servier.com/ (accessed on 25 July 2021).

In line with the large surface generated by the endothelium lining all blood vessels throughout the body, **endothelial-derived MPs** have been found to account for approximately 10% of EVs released into the circulation under physiological conditions [37], and endothelial activation or injury result in an increase of their numbers, as outlined in more detail below. In fact, endothelial-derived MPs (including their cargo) have been suggested as biomarkers of endothelial dysfunction [99]. Endothelial MPs may be distinguished from circulating MPs released from other cellular sources by the expression of endothelial markers, such as CD31, CD105 and CD144, or CD62E and vascular cell adhesion molecule-1 (VCAM-1) in case of endothelial activation [35,100]. As some of the markers, such as CD31, are also expressed on other cell types (e.g., platelets and leucocytes), the ISEV guidelines recommend the use of several (three or more) markers, including at least one transmembrane/lipid-bound protein (e.g., tetraspanin, integrin) and one cytosolic protein (e.g., ALIX, TSG101), exclusion of antigens expressed by other cell types and the use of negative markers (e.g., AGO2) to characterize any EV preparation [16,17] and to differentiate it from other, non-vesicular extracellular matter [101]. A comprehensive summary of cell-specific EV markers is provided in Figure 2. Typical contaminants of EV preparations, such as heat-shock proteins, tubulins, actins, elongation factors or histones, are reported in [102].

Generation of EVs from endothelial cells is reflective upon the type of endothelial injury that caused the process. Specifically, abnormal activation of endothelial cells versus apoptosis causes the externalization of different surface molecules: high levels of the surface antigens E-selectin, ICAM-1, VCAM-1 and other proinflammatory mediators were shown to be expressed on MPs shed from activated endothelial cells [103], whereas low levels of some of these antigens were found on MPs derived from endothelial cells undergoing apoptosis [39]. These studies also revealed that MPs can transfer their inflammatory state to recipient cells and induce functional changes [103].

MPs released from all types of activated or apoptotic cells, including activated endothelial cells, or at least subfractions thereof, are characterized by the presence of PS [100], an anionic phospholipid strictly localized on the inner leaflets of the cell membrane and externalized to the outer surface only during cellular activation or apoptosis [15]. Exposure of TF on endothelial cells is another mechanism underlying the procoagulant activity in response to stimulation with proinflammatory stimuli, as shown for TNFα [40,57] or interleukin (IL) 1α [35]. Higher levels of circulating TF-positive endothelial MPs have been described in blood of patients presenting with antiphospholipid antibodies with lupus anticoagulants activity, particularly in those with a thrombotic complication in the past [37]. Moreover, they may disseminate their procoagulant and proinflammatory potential by interacting with immune cells, as shown in THP1 monocytic cells [41]. An increased exposure of PS or TF on MPs has been associated with hypercoagulability and thrombotic complications, for example in patients with retinal vein occlusion [104] or overweight and obesity [105]. Of note, endothelial-derived MPs are also harboring active tissue factor pathway inhibitor (TFPI) that limits abnormal activation and expression of TF [106]. Notably, thrombin itself may also trigger EV formation, and activation of protease-activated receptor (PAR)-2 by agonist peptide was shown to promote the release of TF-positive endothelial EVs with high procoagulant activity, whereas activation by FVII caused integrin β1-mediated TF internalization [107]. In addition to TF, a variety of adhesion proteins, metabolic enzymes and other antigens were found to be regulated in MPs shed from TNFα-stimulated human umbilical vein endothelial cells (HUVECs) and to stimulate clotting of factor VII-deficient human plasma [82]. Of note, during thrombosis and hemostasis the diverse cell types interact and influence each other, including activation and EV formation. For example, endothelial activation, induced by flow or TNFα, significantly increased the expression of CD62P and TF on MPs within whole blood [108].

MPs may also support plasmin generation and thus promote fibrinolysis [109], adding to the role of blood-borne factors in the equilibrium of thrombus generation and resolution. For example, MPs isolated from TNFα-stimulated endothelial cells were found to expose increased levels of the urokinase type plasminogen activator receptor (uPAR) on their surface and to effectively convert plasminogen into plasmin [43], which may enhance their ability to degrade fibrin clots and to promote vascular wound healing processes. However, only MPs generated and released from endothelial cells and leucocytes, but not those from platelets or erythrocytes, were found to possess the ability to enhance fibrino-lysis through the above-mentioned uPAR-dependent mechanism [110].

**Platelet-derived EVs** constitute another class of cell-specific EVs that are deeply involved in hemostasis and the onset and progression of thrombotic processes. Indirect proof for a role of platelet-EVs in hemostasis came from observations that genetic disorders associated with the inability to shed membrane MPs, such as Castaman defect [111], Glanzmann’s thrombasthenia [112] or Scott syndrome [113], are associated with bleeding diathesis.

Platelet EVs represent the most abundant type of cell-specific EVs circulating in human blood [18,114]. In addition to platelet identification markers, such as CD41 (integrin αIIb) or CD42b (glycoprotein, GpIb), platelet-specific activation markers, such as CD62P (P-selectin) or the fibrinogen receptor integrin αIIbβ3 can be detected. Cryo-electron microscopy and immuno-gold labeling revealed that approximately 30% of all EVs in platelet-free plasma stain positive for CD41 [15]. More unspecific markers include CD31 and CD61 (integrin β3), both of which are also expressed on endothelial EVs, or CD63, a membrane tetraspanin expressed on all EVs. Of note, platelet membrane-derived EVs have to be distinguished from intracellular platelet granules and their contents, which also are released into the microenvironment upon activation as a mode of intercellular communication. Megakaryocyte-derived MPs can be discriminated from platelet-MP by the expression of filamin-A [115]. Using this marker, it was suggested that the majority of circulating MPs in mice and healthy humans are of megakaryocyte origin [115].

EVs have been shown to be formed and released from platelets activated by diverse agonists, including adenosine diphosphate (ADP) [116], thrombin [45,52,53,117], and collagen [118], alone or in combination [52], the thrombin receptor agonist peptide SFLLRN (TRAP) [117], but also exposure to activated endothelial cells under flow [108] or artificial surfaces [112]. Strenuous blood flow conditions, such as occurring in arteries with severe stenosis, may also promote the formation of platelet-MPs [119,120]. Interestingly, the platelet agonist epinephrine was found not to stimulate platelet MP release, despite its effects on platelet aggregation [119]. Platelet antagonists, such as aspirin [121,122] or the adenosine P2Y12 ADP receptor antagonists clopidogrel [123,124] or tigracrelor [125] significantly reduced the release of procoagulant EVs (MPs and exosomes) from activated platelets. Others found direct inhibitors of thrombin (dabigatran and melagatran) to reduce the number of TF-expressing MPs in response to ADP or thrombin stimulation [51]. Notably, monoclonal antibodies against GPIbα suppressed prothrombotic platelet-MP formation under high shear more effectively than the GPIIb/IIIa antagonist abciximab [126].

Electron microscopy, NTA and mass spectrometric proteome analysis revealed that not only the donor, but also the agonist type and pathway activated to stimulate platelet-MP release affects their numbers, size distribution and cargo [45,127,128]. Similar to endothelial cells, different triggers were shown to selectively stimulate the exposure of markers of apoptosis (that is PS) and activation (that is CD62P) on the platelet surface [50]. Earlier studies have shown that exposure of platelets to collagen, but not to fibrinogen, exposes procoagulant phospholipids and promotes their ability to convert prothrombin into thrombin [129]. Analyses in rabbits using biotin-labeled platelet-EVs revealed that they are rapidly (within 10 min) cleared from the circulation and do not reappear within the next 50 min, suggesting continuous generation in states of elevated circulating MP levels [130]. Short half-lives (5–6 h) of EVs carrying the platelet marker CD61 or binding annexin V, CD62P or CD63 were also reported in humans after being transfused into severely thrombocytopenic patients [131].

Whereas the majority of platelet-derived EVs in unstimulated human plasma does not bind annexin V [33], agonist-stimulated platelet-EVs expose procoagulant and proinflammatory PS increasing their ability to bind coagulation factors VIIIa and Va [117]. The procoagulant activity of one platelet-EV was shown to almost equal that of one platelet [132], suggesting that platelet-EV formation during activation represents an effective way of multiplying the thrombotic response to activation. The dose-dependent effects of platelet-EVs on thrombin generation could be almost completely blocked by annexin V (to mask PS) or corn trypsin inhibitor (to block factor XIIa), whereas no effects were seen after blocking with anti-tissue factor antibodies [55]. Others found that platelet- and erythrocyte-derived MPs failed to induce coagulation in factor XII-deficient plasma [133]. In addition, EVs isolated from healthy human blood were found to support coagulation in vitro via TF-independent pathways, at least in part involving factors FXII, XI, IX and VIII [18,134]. Although PS exposure alone may not be sufficient to initiate thrombosis, it may lower the threshold for activation by additional, more potent stimuli and facilitate the interaction of different cell types involved in thrombus formation. In line, experimental studies demonstrated that platelet-EVs support thrombin generation only in the presence of a procoagulant stimulus [135], such as TF exposed on monocytes or the vessel wall after injury. Others found circulating CD42b-positive MVs shed from activated platelets to be the major source of TF present in plasma [136]. Notably, in vitro studies found that EVs derived from platelets not only bind annexin V or lactadherin, but also protein S, a co-factor of activated protein C, indirectly suggesting that MPs also have the potential to support anticoagulant reactions [137].

In addition to direct effects on the coagulation cascade, activated platelets may contribute to thrombosis and link this event with inflammation. For example, high-shear stress induced platelet-MPs were shown to induce the expression of adhesion molecules on endothelial cells [138], and deposition of the chemokine RANTES by MPs generated from activated platelets promoted monocyte arrest to inflamed and atherosclerotic endothelium [139]. The rapid adhesion of platelet-derived vesicles to monocytes was shown to occur via P-selectin resulting in the transfer of platelet GPIbα to monocytes and enabling their interaction with additional ligands, such as vWF [140]. Thus, platelet MPs may deposit and transfer their specific receptors to the surface of other cells and thereby aid in amplifying the coagulation process. In line, platelet-derived MPs were shown to enhance platelet adhesion and fibrin deposition on human atherosclerotic arteries ex vivo perfused with human whole blood [87].

Immune cells, in particular **monocytes** and granulocytes, participate in the early phases of thrombus formation by interacting with platelets and endothelial cells to promote thrombin generation [141]. Activation of the coagulation cascade is achieved by overexpression of active TF on inflamed blood (and endothelial) cells. Interestingly, kinetic analyses in living mice suggested that the majority of TF accumulating during the initial phases of arterial thrombus formation originates from microparticles, and not from circulating leucocytes, which are recruited later [142]. An integral hemostasis test revealed that ability to induce spontaneous clotting was highest for monocyte-MPs and mediated through the TF pathway, whereas platelet or erythrocyte MPs activated clotting through the contact pathway only [97]. Whereas in unstimulated whole blood neither monocytes nor platelets were found to express detectable amounts of active TF, LPS stimulation increased the number of monocytes, but not of platelets expressing TF [56]. Several studies have shown that the majority of MPs expressing TF are derived from monocytes [85,98,133], whereas MPs from platelets did not support factor Xa generation and exhibit lower prothrombinase activities [135]. Direct comparison of the thrombogenicity of platelet and monocytic cell-derived EVs revealed that platelet-EVs do not express TF, whereas LPS stimulation increased TF expression and thrombin generation in monocytic EVs by approximately 10-fold [55]. In addition, monocyte-MPs may promote fibrin formation and increase fibrin network density, thus promoting blood clot stability [135]. Findings from this and other studies [98] suggested that all EVs support the propagation of coagulation via exposure of PS, while expression of functional TF on EVs appears to be limited to pathological conditions [55]. In line, monocytic EVs failed to support FXII-dependent thrombin generation when added to EV-depleted human plasma, in contrast to EVs originating from platelets or erythrocytes [143]. Interestingly, enrichment of human monocytes/macrophages with cholesterol induces PS exposure and TF-positive MP release [144], linking hyperlipidemia with an increased risk of atherothrombosis. With regard to patients with arterial thrombosis, the amount of TF expressed on monocytes was significantly elevated in patients with ACS [145,146], and higher numbers of circulating TF-positive MPs were detected in the occluded coronary artery compared to peripheral blood [147], in line with their enhanced recruitment and consumption during thrombus formation and accumulation at the site of injury.

EVs have the capability to promote thrombosis also through indirect mechanisms, that is by activating and enhancing the procoagulant properties of other cell types, such as platelets and endothelial cells. Real-time in vivo microscopy revealed that the accumulation of monocyte-derived MPs expressing TF and PSGL-1 in human and murine thrombi depends on their interaction with CD62/P-selectin-positive platelets [94]. MPs and exosomes released from THP1 monocytic cells following activation by starvation, LPS or the calcium ionophore A23187 were shown to increase the thrombogenicity of endothelial cells, whereas levels of the endothelial anticoagulants tissue factor pathway inhibitor (TFPI) and thrombomodulin decreased [54]. Conversely, MPs derived from TNFα-activated endothelial cells were found to interact with THP1 monocytic cells and to enhance their procoagulant properties, which involved increased expression of TF on monocytes [41].

In addition to endothelial cells, platelets and white blood cells, EVs may also be generated from **erythrocytes** (red blood cells; RBCs), in particular from the membrane of aged or senescent erythrocytes [148]. RBC-derived EVs are found in high concentrations in erythrocyte concentrates, in particular after prolonged storage and following harsh processing methods [149], and are believed to be responsible for both beneficial and detrimental effects observed following blood transfusions [150]. Similar to platelets [137], they were shown to expose PS and bind lactadherin and annexin V and to assemble tenase and prothrombinase complex formation thus supporting thrombin generation, both in the presence and absence of added TF [151]. Others found RBC-derived MPs from expired stored erythrocyte concentrates to increase the expression of TF on monocytes in whole blood from healthy volunteers and to promote platelet-monocyte aggregate formation [152]. However, and despite elevated total EV numbers, transfusion of stored autologous RBCs into healthy volunteers did not further enhance thrombin generation elicited by LPS [153].

Although the mechanisms of EV formation from RBCs have not been fully defined, they involve a complement-mediated influx and rise in intracellular calcium as well as a calcium-independent increase in oxidative stress [154], among possible other, unidentified pathways. Malaria infection [155] or (inherited or acquired) hemoglobinopathies [156] also may promote the formation of procoagulant MPs from erythrocytes and mediate the prothrombotic risk associated with these conditions [157].

Increased levels of CD235a-positive, erythrocyte-EVs were observed in growing thrombi following ex vivo exposure of blood to extracellular matrices mimicking damaged arterial wall components [158]. Similar to platelets, MPs derived from activated erythrocytes were shown to trigger thrombin generation via factor XIIa and to potentiate contact factor pathway-mediated coagulation [133]. RBC-MPs may initiate coagulation also by directly activating factor XII or pre-kallikrein leading to factor IX activation and thrombin generation [159].

## 6. EVs and Thrombosis in the Arterial System: Clinical Evidence

Activation of endothelial or circulating blood cells resulting in procoagulant MP formation has been suggested to underlie the increased thrombotic risk associated with diverse clinical conditions, such as metabolic syndrome [160], coronary heart disease [161], atrial fibrillation [162] or vascular stent implantation [163,164].

In line with endothelial dysfunction and increased platelet activation during myocardial infarction (MI), several studies found increased circulating numbers of circulating MPs in patients with ACS. In one of the first studies, elevated levels of annexin V-positive, procoagulant MPs were observed in circulating blood of patients with ACS (unstable angina, MI) and found to be higher than those in patients with stable coronary artery disease (CAD) or chronic non-coronary heart disease [27]. Capture with antibodies directed against cell type-specific antigens revealed that the majority of MPs were positive for CD31 and CD146 suggesting endothelial origin, whereas those captured with anti-GPIb, anti-CD3 or anti-CD11a antibodies did not differ. Similar findings were obtained in a prospective, case-controlled study including 84 patients with CAD and 42 control subjects [165]. Elevated numbers of CD31-positive, CD42b-negative MPs were found to discriminate patients with ACS from those with stable angina [166]. Interestingly, platelet-EV numbers did not differ in those studies, although a correlation between CD31-positive endothelial EVs and platelet-EVs was observed [27,165], potentially also due to the overlap of antigen expression on the surface of both cell types, as mentioned earlier. Elevated levels of circulating endothelial and platelet-EVs were primarily observed during the acute thrombotic event [167]. A systematic review and meta-analysis of 11 observational studies performed before 2015 revealed significant differences in plasma platelet-EV concentrations between patients with ACS and healthy controls, whereas their numbers did not differ compared to patients with stable angina [168]. More recently, a meta-analysis of case–control studies revealed higher levels of EVs, including CD31-positive, CD42-negative and CD144-positive endothelial EVs, in patients with coronary heart disease (CHD) compared to controls, and their numbers were also higher in patients with MI vs. unstable angina vs. stable angina [166].

Not only the total number, but also the cell-specific origin of circulating EVs was found to change over time, and higher numbers of circulating EVs with a characteristic signature (CD66b+/CD62E+/CD142+) were observed at early time points following acute myocardial ischemia [169]. Time course analysis revealed markedly elevated circulating levels of PS-positive procoagulant MPs, primarily of those expressing the platelet marker GPIb, and to a lesser extent of those positive for the endothelial marker CD31, at day 0 in patients during STEMI and unstable angina, which subsequently decreased following treatment (i.e., percutaneous coronary angioplasty (PTCA) and GPIIbIIIa inhibitors), but still were significantly elevated at day 6 after the initial event [170]. Similar findings were obtained for plasma soluble glycoprotein V levels, a marker of platelet activation [171]. Others found higher CD41-positive platelet-MP numbers only in patients with non-ST-elevation MI and time-dependent changes (over a two-year follow-up) only for CD41-positive platelet MPs, whereas endothelial or monocyte MP levels did not differ during this time period [172]. The number of CD62P-positive procoagulant MPs was found to increase during follow-up over two years and to be associated with an adverse long-term cardiovascular outcome [172]. Interestingly, lower numbers of circulating CD62P+/TF+ MPs were observed in patients with atherosclerosis in several arterial beds (i.e., coronary, carotid and peripheral vasculature) [172], possibly due to their increased CD62P–PSGL1 mediated adherence to the vessel wall, as observed in another experimental study [94]. Others found lower plasma concentrations of EVs from activated platelets (CD61+/CD62P+) and platelet-EV–fibrinogen aggregates in patients with acute MI treated with the P2Y12 antagonist ticagrelor for 6 months compared to those treated with clopidogrel, whereas the number of endothelial-EVs (CD31+/CD146+) did not change [173]. Ticagrelor inhibits platelet aggregation also by increasing the anti-platelet and anti-inflammatory agent adenosine [170], which may explain its more pronounced effect on platelet-EV release [125]. In patients with stable CAD 12 months after percutaneous coronary intervention (PCI) and stent implantation P2Y12 inhibitor intake was found not to have any effects on the levels of PS-positive EVs or EV subpopulations from platelets, erythrocytes, monocytes or endothelial cells [174]. That the levels of coronary EVs may also reflect the severity of injury is suggested by findings that their numbers inversely correlated with the time elapsed until intervention and the duration of ischemia [169].

Local EV accumulation and action is suggested by findings that platelet procoagulant activity and platelet-MP numbers are significantly elevated in the coronary artery compared to venous blood of patients with unstable angina, before and after PTCA and stent implantation, whereas platelet reactivity was not affected by the procedure [175]. Increased levels of platelet-MPs and other platelet activation markers (e.g., P-selectin expression, platelet–monocyte heterotypic aggregate formation) were observed as early as 15 min after stenting the occluded coronary arteries [176]. Further analyses revealed that platelet-MPs are more strongly induced and also detectable for longer than conventional markers of platelet activation [175]. Thrombogenic MPs were also detected in human atherosclerotic plaques and found to primarily originate from monocytes/macrophages and lymphocytes, but not from platelets [177,178]. Importantly, plaque-MPs almost completely retain their total TF activity [177]. Suggesting that local MP generation may contribute to the formation of intracoronary thrombi, levels of leukocyte-derived CD11a-positive MPs, endothelial-derived CD105-positive MPs and TF-bearing MPs were significantly higher within the occluded coronary artery compared to peripheral blood collected from a distant site (femoral artery) of 12 patients with ST-segment elevation myocardial infarction (STEMI) and decreased following restoration of coronary blood flow [147]. A similar local increase in MPs was seen by others in aspirates of culprit coronary arteries in patients with STEMI compared to levels in the femoral artery of the same patient [179]. Levels of intracoronary endothelial and platelet MPs were also found to be higher locally than in the systemic circulation (aorta) in 78 STEMI patients undergoing percutaneous coronary intervention (PCI) and found to correlate with thrombus score [180]. An independent correlation of local platelet-MP levels with electrocardiographic and angiographic indices of microvascular obstruction was also observed in this study. Others found endothelial MPs in plasma to be 2.5-fold higher in patients presenting with coronary lesions angiographically scored as eccentric or high-risk and 3-fold higher if lesions with thrombi were present [181]. The results of these studies strongly suggest that circulating MP levels may reflect complications of atherosclerosis, that is plaque rupture and thrombosis. Proteome analysis of plasma MPs from STEMI patients and stable ACS controls revealed differential protein expression patterns and upregulating of factors involved in thrombogenesis [182], indicating that circulating MP levels not only reflect injury or activation of the cell of their origin, but that they actively participate in atherothrombosis.

Higher numbers of MPs and PS-positive cells were also observed in the circulation of patients with non-ST elevation myocardial infarction compared to healthy controls already at baseline, and their levels further increased following stent implantation [163]. An increase in PS expression on platelets and erythrocytes was detected as early as one hour following stent implantation and peaked at 18 h [163]. The contribution of PS to the hypercoagulability following non-STEMI was demonstrated by the finding that lactadherin successfully blocked the MP-mediated procoagulant activity and fibrin formation, whereas antibodies against TF had no effect [163]. Lactadherin is a small, secreted extracellular matrix protein known to bind PS-enriched cell surfaces in a receptor-independent manner [183], which may block the procoagulant activity of platelets and thrombosis [34].

Regarding the value of EVs as biomarker for risk prediction and outcomes, circulating levels of CD31-positive, annexin V-positive MPs may also be useful as independent predictors of cardiovascular outcomes, as suggested by findings in 200 patients with stable CAD followed for a mean of 6 years [184]. Intracoronary levels of CD144-positive endothelial MPs were also found to be higher in patients with sudden cardiac death due to acute coronary occlusion and ventricular arrhythmia compared to patients with STEMI without rhythmic disturbance, whereas intracoronary and systemic blood concentrations of platelet-derived MPs did not differ [185]. Almost 2-fold elevated levels of MPs derived from erythrocytes, but no changes in platelet-MP numbers, were observed in patients with STEMI after primary coronary intervention (PCI), independent of total RBC numbers, compared to age-matched cohort of healthy volunteers [186]. Multivariate regression analysis revealed that erythrocyte-MP numbers were independently associated with the occurrence of the composite clinical endpoint within the 6-months follow-up after the index event [186]. Although the numbers of small-size MPs positive for endothelial cell marker CD144 did not differ in patients with non-STEMI and CAD controls, their numbers were found to be independently predictive for future admissions related to heart failure [187].

Similar to the above findings in patients with thrombotic occlusion of the coronary artery, thrombosis or thromboembolism of the cerebral vasculature also was found to be associated with increased circulating numbers of prothrombotic EVs. For example, higher CD62E-positive endothelial EV levels were observed in a prospective study examining a total of 348 consecutive patients with acute stroke (*n* = 73) compared to patients with vascular risk factors but no stroke event (*n* = 275) [188]. The authors also found CD62E-positive MP levels to be associated with recent ischemic episodes and larger infarct volumes, and distinct endothelial MP patterns were observed in patients with extracranial and those with intracranial arterial stenosis [188].

Elevated numbers of MPs expressing PS were observed in patients with heart failure, a prothrombotic state frequently associated with endothelial dysfunction, and shortened coagulation times and increased factor Xa/thrombin generation could be blocked (by approximately 80%) using lactadherin [189]. Increased numbers of circulating TF-positive procoagulant MPs, in particular those carrying the monocyte marker CD14, were also observed in patients with STEMI, who later developed heart failure [190] or died because of cardiovascular causes during the study follow-up [191]. Preliminary analysis in a small number of patients with end-stage heart failure and implanted left ventricular assist device suggest that upregulation of PS-positive, procoagulant MPs may be useful biomarker of adverse, thrombotic events [192].

## 7. EVs and Thrombosis in the Venous System: Findings in Human Studies

Regarding thrombus formation in the venous system, several studies have shown that circulating levels of procoagulant MPs are elevated in patients suffering from deep vein thrombosis (DVT). For example, higher levels of TF-positive MPs were observed in patients with acute pulmonary embolism (PE) compared to healthy controls [193,194]. Elevated numbers of monocyte-derived TF-positive MPs and a higher procoagulant activity of platelet-free plasma were detected in patients with initial or recurrent DVT compared to sex- and age-matched healthy controls [195]. Other groups report low levels of TF-positive MPs in a small group of patients with unprovoked DVT, both in the acute phase and during follow-up for 6 months [196]. Increased numbers of circulating TF-positive or annexin V-positive MPs derived from endothelial cells or platelets and shorter clotting times were also observed in carriers of a common prothrombin gene mutation (G20210A) [197] or factor V Leiden (FVL) gene mutations [198], known to predispose to venous thromboembolism. MP numbers did not differ in FVL carriers with and without a history of thrombosis [199], whereas significantly higher levels of annexin V-positive MPs and shorter clotting times were observed in those with a previous history of venous thromboembolism (VTE) [198].

The number of circulating total MPs, together with plasma D-dimer and soluble P-selectin levels, was found to be higher in symptomatic patients with DVT and positive Duplex ultrasound [200]. Circulating total EV numbers were also higher in patients with VTE, and the VTE risk was found to increase progressively with increasing MP numbers, independent of other risk factors associated with a hypercoagulable state [201]. Elevated CD62P-positive platelet MPs were observed in patients with unprovoked DVT [202], and their numbers also were elevated in patients with acute PE, whereas endothelial MP levels did not differ [193,194]. Others report elevations in circulating endothelial MP numbers, also in conjunction with monocytes, in patients with acute venous thromboembolism (VTE) [203]. In contrast, circulating PS-positive MP numbers in patients with a history of recurrent VTE did not differ from those in controls, including different subgroups, and no association of circulating MP numbers with VTE was found [204].

Many studies have examined the role of EVs as potential risk factors for DVT in patients with cancer. In line with EVs being released from activated or apoptotic cells and expressing TF or negatively charged phospholipids to promote coagulation, the risk of developing venous thrombosis is significantly higher in patients with cancer [205]. However, and although higher numbers of TF-positive MPs carrying endothelial and platelet markers were observed in plasma of patients with active cancer (with and without VTE), multivariate analysis failed to show a significant association between their numbers and the presence of thrombosis [206]. Similarly, a prospective cohort study found the activity of TF on MPs in cancer patients to be independently associated with mortality and prognosis, but not with thrombosis [207]. Elevated levels of fibrin-bearing MPs have been described as potential biomarkers of thrombo-embolic events in patients with colorectal and pancreatic cancers and were found to be associated with reduced survival [184]. Interestingly, this MP subtype was more frequently produced by erythrocytes, endothelial cells or Ep-CAM-positive cells than by platelets or leukocytes. For more details and insights into the association between EVs and cancer-associated thrombosis, the reader is referred to recent excellent review articles on this topic [208,209,210].

## 8. Extracellular Vesicles and Thrombosis Associated with COVID-19 Infection

COVID-19 (Corona Virus Disease-19) is a viral infectious disease that emerged in 2019 and is caused by a novel coronavirus called Severe Acute Respiratory Syndrome Corona Virus-2 (SARS-CoV-2). SARS-CoV-2 primarily targets epithelial cells of the respiratory tract to enter the host organism leading to symptoms ranging from partial or total loss of olfaction to the acute respiratory syndrome. The local and systemic inflammation and overactivation of the immune system together with endothelial dysfunction and platelet activation generate a systemic prothrombotic state in patients with COVID-19. Therefore, it comes as no surprise that microthrombosis in diverse vascular beds followed by multi-organ damage and failure, but also pulmonary embolism or stroke are among the major complications and potential causes of death in patients infected with SARS-CoV-2 [211,212]. For more details on the mechanisms underlying the coagulopathy in COVID-19 and its therapeutic management, the reader is referred to excellent review articles dedicated to this topic [213,214].

Importantly, the presence of fragmented SARS-CoV-2 viral genome or proteins in platelets and endothelial cells, but possibly also other cell types involved in thrombosis and hemostasis, suggests that these cells also are direct targets of the virus and that the resulting cellular activation, dysfunction and death contributes to thrombosis in COVID-19 [214,215,216,217]. As in other forms of cellular activation, the release of procoagulant EVs also may play a significant role in the micro- and macrovascular thrombotic complications associated with COVID-19.

Elevated EV numbers have been reported in severely ill, hospitalized patients infected with SARS-CoV-2 [218]. Others found elevated circulating numbers of CD41^+^CD31^+^ platelet EVs to predict the presence of SARS-CoV-2 infection [219]. Notably, increased circulating EV numbers were already detectable in asymptomatic subjects diagnosed with COVID-19 [220]. Similar to other procoagulant states associated with endothelial or platelet activation, as outlined above, EVs in COVID-19 patients were shown to carry increased levels of biologically active TF [218,220,221] or PS [215] on their surface. However, and in contrast to patients with septic shock not related to COVID-19, TF-EV levels were markedly higher and paralleled by increased fibrinolytic activities not counterbalanced by increased PAI-1 [218], in line with the existence of a COVID-19 specific coagulopathy [212].

In addition to observations of elevated circulating EV numbers and their associations with disease severity, first studies reported a specific surface antigen signature [220] or protein cargo [222] in circulating EVs isolated from patients diagnosed with COVID-19. It also was speculated that changes in EV cargo in subgroups of COVID-19 patients, such as those with diabetes mellitus, obesity or hypertension, may contribute to the increased morbidity and mortality observed in these high-risk populations [223]. Importantly, addition of plasma from severely-ill patients with COVID-19 to endothelial cells ex vivo was found to transfer a prothrombotic phenotype and to induce apoptotic cell death [222] suggesting the presence of a vicious cycle between endothelial injury and EV release in COVID-19 associated thrombosis.

Future studies in this direction will shed more light on the specific contribution of EVs to the coagulopathy in COVID-19 or other viral infections associated with an increased risk of thrombosis [224] and also clarify their role as mediators of thromboinflammation, biomarkers of disease severity or therapeutic vehicles. Please also see the review article by Adriana Georgescu and Maya Simionescu for more details [225].

## 9. Arterial and Venous Thrombosis: Findings in Mouse Models

Observations in experimental models of arterial and venous thrombosis have shed further light on the role and contribution of EVs in the pathogenesis of thrombus formation under in vivo conditions and in the presence of risk factors. Falati et al. have shown that MPs exposing TF on their surface participate in fibrin generation in a laser-induced mouse model of arterial thrombosis [94]. The interaction of TF-positive EVs with thrombi was suggested to occur through the interaction of PSGL-1 expressed on MPs and P-selectin expressed on platelets [94]. This was later confirmed by experiments showing a reduced accumulation of TF-positive MPs at the site of thrombosis in mice lacking P-selectin or PSGL-1 [226]. Ghosh et al. reported that binding of MPs to the PS receptor CD36 expressed on platelets sensitized them to further agonist stimulation resulting in increased platelet activation and enhanced arterial thrombus formation [227]. MPs isolated from stored platelet concentrates were shown to adhere following wire-induced vascular injury in Swiss mice, and platelet adherence could be reduced, albeit not completely prevented, using antibody fragments directed against integrin αIIbβ3 [228]. Others have shown that MPs generated from activated platelets significantly shortened the time to arterial thrombosis in mice following carotid injury (induced by Rose Bengal) and found that this occurs via transfer of miR-223 to endothelial cells and downregulation of insulin-like growth factor-1 receptor [229].

Regarding thrombus formation in the venous system, experimental studies using the inferior *Vena cava* (IVC) ligation model could correlate changes in the number or activation of EVs derived from platelets, leucocytes and endothelial cells with the extent of venous thrombosis. Mechanisms of EV generation during venous thrombosis may include increased oxidative stress [230] or apoptotic cell death and senescence [231]. Time course analysis following experimental induction of venous thrombosis in wild-type mice revealed a positive correlation between venous thrombus weight and the number of platelet-derived MPs, whereas a negative correlation was observed for leucocyte-MPs [232]. The association between platelet-MPs and venous thrombus weight was also observed in mice lacking P- or E-selectin. Studies in knockout mice support the role of P-selectin in venous thrombus formation [233], which was shown to involve P-selectin on platelet-, but not on leucocyte-derived MPs [234]. In this regard, P-selectin expressed on platelets (and presumably also on MPs released from them) was shown to rapidly trigger the exposure of TF on monocytes in response to platelet activators [235]. Importantly, PAR1 agonist peptide-triggered monocyte TF exposure required the presence of platelets, P-selectin and PSGL-1, but only if coupled to 2 µm beads mimicking EVs. The role of TF on MPs for venous thrombus formation in vivo was confirmed in mice injected with MPs isolated from IL1α-stimulated HUVECs [36]. Using a rat model of venous stasis, Birò et al. could show that TF-positive MPs isolated from pericardial blood of cardiac surgery patients were highly thrombogenic compared to microvesicles isolated from healthy subjects, and MP thrombogenicity in vitro could be abolished using inhibitory antibodies against TF [93].

Regarding the mechanisms underlying the increased risk of venous thrombosis in some forms of cancer and the role of EVs therein, it was observed that MPs isolated from TF-overexpressing A2780 (ovarian cancer) cells when injected into operated mice enhanced venous thrombus formation and clot burden following experimental IVC stenosis [236]. Findings that mice with TF-positive tumors and elevated levels of circulating TF-positive EVs exhibited increased thrombosis in a saphenous vein model, whereas no differences in thrombus weight were observed between tumor-bearing and control mice in the inferior vena cava stenosis model [237] suggest that the IVC ligation model of venous stasis may not be ideal to examine the role of circulating factors in the pathophysiology of venous thrombosis [238]. That the increased risk of thrombosis, particularly in the venous system, associated with some forms of cancer is linked to TF-positive MPs was confirmed in another experimental study by showing larger clots following IVC ligation in mice bearing human pancreatic tumors compared to controls [239]. Intravital microscopy revealed that MPs derived from pancreatic and lung cancer cells expose TF and PSGL-1, aggregate platelets in a TF-dependent manner and accumulate at the site of injury to promote thrombotic occlusion of venules and arteries [240]. Using a nude xenograft mouse model of human pancreatic cancer, others also could show that cancer cells actively releasing TF-bearing MPs into the circulation have the ability to activate coagulation [237,241]. Others have shown that TF expression on cancer cell-derived MPs plays a critical role in occlusive venous thrombosis, whereas neither P-selectin nor GpIb were required for MP recruitment in this model [242]. TF-positive MPs obtained from human tumor cell lines were shown to activate human platelets and to promote platelet aggregation and venous thrombosis in two mouse models [243]. In this study, EV-triggered venous thrombosis was reduced in mice lacking PAR4 (the thrombin receptor expressed on platelets) or treated with clopidogrel supporting a role for TF-induced platelet activation in this model. Conversely, in mice bearing a tumor under-expressing TF the interaction of cancer cell-derived MPs expressing TFPI with fibrinogen and platelets (via integrin αvβ1 and αvβ3) was associated with decreased bleeding times and shown to hamper activation of the coagulation cascade, platelet aggregation and thrombus formation [244]. Increased exosome-induced formation of neutrophil extracellular traps has also been associated with the prothrombotic state in cancer, as shown in mice orthotopically injected with 4T1 breast cancer cells [245]. A summary of experimental findings on the role of EVs in thrombosis using experimental models is provided in Table 2.

## 10. Conclusions

The putative role of EVs in hemostasis and thrombosis is supported by a large number of clinical and experimental studies unraveling how these cell-derived structures affect clot formation (and resolution). From those studies it has become clear that the prothrombotic effects of EVs are not restricted to the exposure of TF and PS, but also involve multiplication of procoagulant surfaces, cross-linking of different cellular players at the site of injury and transfer of activation signals to other cell types. Their presence in all body fluids, such as blood and urine, makes them an attractive tool for liquid biopsy. Noteworthy, thrombotic disorders are often associated with altered levels of the different classes of EVs, suggesting their potential usefulness as biomarkers of cell-specific injury and pathological processes. Moreover, the number of circulating EVs as well as their protein, RNA or miR content may reflect disease severity and prognosis or predict future adverse events. However, it should be noted that most clinical studies included a relatively low number of subjects and observations must be validated in larger cohorts of patients. Another relevant issue is the limited knowledge about the effect of concurrent use of multiple medications on circulating EV levels. Indeed, it is well known that some antiplatelet drugs, antihypertensive agents and statin therapy may influence shedding and composition of MVs. From a technical point of view, differences in the purification method to isolate EV subtypes, such as differential ultracentrifugation, density gradient centrifugation, size exclusion chromatography, may lead to EV preparations of different composition and purity, which may also affect their function. Therefore, to obtain reliable conclusions on the role of EVs in physiological and pathological conditions or on their use as biomarkers, it is crucial to follow published recommendations or guidelines [16].

Current strategies to prevent or to treat thrombus formation in response to vascular injury focus on the prevention of platelet activation and aggregation, activation of the coagulation cascade or on the restoration of endothelial function. EVs have been demonstrated to play a role in tissue regeneration and given their ability to mediate cell-to-cell communication, EVs may be exploited as a drug delivery system. The ability of EVs to alter the transcriptome and signaling activity in recipient cells allows them to induce specific phenotypic changes. Despite being in its early research stages, discoveries made on the field so far are promising and suggest that EV uptake mechanisms can be manipulated for the design of future therapies. Moreover, they could be genetically modified to produce tailored EVs with increased anti-thrombotic, fibrinolytic or regenerative properties [247]. For example, so-called platelet MP-inspired nanovesicles have successfully been designed to encapsulate thrombolytic drugs and to achieve targeted in vivo fibrinolysis without off-target uptake and action [248]. Others describe a strategy to generate large amounts of EVs from blood group 0 erythrocytes and its usefulness to safely deliver drugs to achieve miR inhibition or genome editing [249]. However, and despite first promising results, the emerging potential of EVs as biomarkers, drug delivery systems or mediators of regenerative mechanisms still needs further exploration to overcome some limitations (concerning cytotoxicity, uptake efficacy, or half-life) on their way towards clinical application [250,251].

In conclusion, advancing our knowledge about mechanisms of EV formation and their pathophysiological relevance may help to shed light on circulating EVs and to translate their application into clinical practice.

## Figures and Tables

**Table 1:** Common cell-specific stimuli of extracellular vesicle formation and analysis.

Stimulus	Cell Type	Concentration/Incubation *	Readout
**Endothelial Cells**			
Proinflammatory stimuli			
IL1α	HUVEC	5 ng/mL 0–72 h	Annexin V binding; TF and endothelial antigen expression; procoagulant effects in vivo [35,36]
IL1β	HUVEC	10 U/mL 24 h	EV release and marker antigen expression [37]
LPS	HAEC	10 ng/mL 24 h	EV release; PS and annexin V expression [38]
PMA	HUVEC	100 ng/mL 24 h	EV release [37]
TNFα	Renal and brain microvascular and coronary artery macrovascular ECs	10 ng/mL 24 h	Marker antigen expression and annexin V binding [39]
TNFα	HUVEC	10 ng/mL 24 h	TF release by small and large EVs [40]
TNFα	HUVEC	1–100 ng/mL 6 h (blebbing) 24 h (vesiculation)	EV release and marker antigen expression; procoagulant activity in vitro [37]
TNFα	HUVEC	100 ng/mL 48 h	MP release [41]
TNFα	HBMEC	20 ng/mL 20 h	MP release [42]
TNFα	HMEC-1	100 ng/mL 48 h	MP release [43]
Procoagulant stimuli			
Ca^2+^ ionophore A23187	HUVEC	100 µmol/L 10 min	EV release [37]
Complement C5b-9	HUVEC	2 µg in 125 µL 10 min	EV release; prothrombinase and thrombomodulin activity [44]
PAI-1	HUVEC	1–10 ng/mL 1–3 h	Procoagulant, uPAR and integrin αvβ3 expression, thrombin generation [44,45,46]
Thrombin	HUVEC	0.1–10 U/mL 24–48 h	Marker antigen expression; procoagulant MP release [37,47,48]
Thrombin	HBMEC	2 U/mL 20 h	Procoagulant MP release [42]
Proapoptotic stimuli			
Serum and growth factor deprivation	Renal and brain microvascular ECs; coronary artery macrovascular ECs	N/A 24 h	Marker antigen expression and annexin V binding [39]
Staurosporine	HUVEC	100 nmol/L 2–16 h	Apoptotic MP/PS exposure; annexin V binding; TF expression; factor Xa activation [49]
**Platelets**			
Ca^2+^ ionophore A23187	Human platelets	10 µM 15 min	CD62P and PS exposure, EV quantity and quality [45,46]
ABT-737	Human platelets	30 µM 90 min	CD62P and PS exposure [50]
ADP	Human platelets	60 µM 30 min	EV quantity and quality [45]
ADP	Human platelets	20 µM 1 h	EV quantity and quality [51]
Collagen	Human platelets	10 µg/mL 30 min	EV generation [45,52]
Complement C5b-9	Human platelets	2 µg in 125 µL 10 min	EV release, factor V binding [7]
CRP-XL	Human platelets	1 µg/mL 30 min	EV generation [45,52]
LPS	Human platelets	100 ng/mL 3 h	EV quantity and quality [45]
Thrombin	Human platelets	1 U/mL 15 min	CD62P and PS exposure [50]
Thrombin	Human platelets	1 U/mL 30 min	EV generation [45,52]
Thrombin and CaCl_2_	Human platelets	0.25 U/mL 5 min	EV quantity and quality [53]
TRAP6	Human platelets	60 µM 30 min	EV quantity and quality [45]
**Monocytes**			
Ca^2+^ ionophore A23187	THP-1	10 mM 5 min	TF^+^ MP release [37,54]
LPS	THP-1	5 µg/mL 4 h	MP release [55]
LPS	THP-1	1 µg/mL 1 h (RT)	MP release [56]
LPS	THP-1	1 µg/mL 5 h (RT)	TF^+^ MP release [54]

* Incubation at 37 °C, if not stated otherwise.

**Table 2:** Findings in animal models of thrombosis.

EV Type	Model	Species	Main Findings
**Arterial thrombosis**			
MPs	Laser-induced endothelial injury of arterioles in cremaster muscle	C57BL/6J, B6.Cg-SelPltm1Fur and C57BL/6J-Selptm1Bay mice	Interaction of TF-positive EVs with thrombi occurs through the interaction of PSGL-1 expressed on microparticles and P-selectin expressed on platelets [94]
MPs	FeCl_3_-induced injury of carotid artery	C57BL/6J and CD36^−/−^ mice	PS-exposing MPs released from activated platelets bind to CD36 receptor on platelets which results in enhanced platelet activation and thrombus formation [227]
MPs	Wire-induced endothelial denudation of carotid artery	Swiss mice	MPs isolated from stored murine platelet concentrates adhere on the vascular wall through an integrin aIIbβ3-mediated mechanism [94]
EVs	Rose Bengal-induced carotid artery injury	C57BL/6J and miR-223^−/−^ mice	Platelet-derived EVs accelerate arterial thrombus formation by transferring miR-223 and downregulating IGFR-1 on endothelial cells [229]
Tumor exosomes	Tandem stenosis at the carotid artery	C57BL/6J WT and apoE^−/−^ mice	Elevated plasma exosomal miR-223, miR-339 and miR-21 levels in mice following induction of atherothrombosis [246]
**Venous thrombosis**			
MPs	IVC ligation	C57BL/6J, CD62E^−/−^, CD62P^−/−^ and Delta Cytoplasmic Tail mice	MPs derived from platelets and leukocytes correlate with venous thrombus weight MP re-injection leads to higher thrombus weight than IVC alone, TF on MPs correlates to MP numbers [232]
MPs	IVC ligation	C57BL/6, CD62P^−/−^ and CD62E^−/−^ mice	An association between platelet-MPs and venous thrombus weight was observed in mice lacking P- or E-selectin [233]
MPs	IVC ligation	C57BL/6J, CD62E^−/−^, CD62P^−/−^ and Delta Cytoplasmic Tail mice	High circulating levels of soluble P-selectin and leucocyte MPs are associated with increased venous thrombosis. Antibodies directed against PSGL-1 decreased thrombus mass [234]
MPs	IVC ligation/stasis	Wistar Hsd/Cpb; WU rats	Human cell-derived microparticles promote venous thrombus formation in a tissue factor-dependent manner [93]
MPs	IVC ligation/stasis	Rats	MPs from IL1α-stimulated endothelial MPs promote venous thrombus formation, and thrombus weight is significantly reduced by anti-TF, but not by anti-Factor XII antibodies [36]
TF^+^ tumor EVs	IVC ligation/stenosis	Nude mice	EVs from TF-overexpressing human ovarian cancer cells enhance murine venous thrombus formation [236]
TF^+^ tumor MPs	IVC ligation/stenosis	Nude mice	Thrombus weight does not differ between human pancreatic tumor-bearing and control mice, whereas injection of TF^+^ MPs dose-dependently increases venous thrombosis [237]
TF^+^ tumor MPs	IVC ligation	Nude mice	Venous clots are larger in mice bearing tumors compared to controls [239]
Tumor MPs	Laser and FeCl3 injury, cremaster and mesentery	C57BL/6J mice	Mice with tumors have larger thrombi, and infusion of a blocking P-selectin antibody abolishes the thrombotic state observed after injection of MPs or in mice developing a tumor [240]
Tumor MPs	IVC ligation/particle flow restriction	C57BL/6J mice	Larger venous thrombi in tumor-bearing mice or mice injected with high TF tumor MPs having more pronounced effects than low TF tumor MPs [240]
TF^+^ tumor MPs	Femoral and IVC ligation	C57BL/6J and PAR4^−/−^ mice	TF-positive MPs obtained from human pancreatic adenocarcinoma expressing low or high TF levels enhance venous thrombosis in two mouse models. Reduced thrombosis in PAR4^−/−^ mice and in mice treated with clopidogrel suggested role of platelets [243]
TF^+^ tumor MPs	Laser injury cremaster muscle	C57BL/6J mice	Inhibition of platelet activation reduces integrin αvβ1 and αvβ3 dependent accumulation of cancer cell MPs and thrombosis in human pancreatic tumor-bearing mice [244]
Tumor exosomes	Rose Bengal venous thrombosis	BALB/c mice	Intravenous administration of 4T1-derived exosomes enhances NET formation and venous thrombosis in mice treated with G-CSF [245]

## Abbreviations

ACS	acute coronary syndrome
ADP	adenosine diphosphate
AMI	acute myocardial infarction
ANG II	angiotensin II
Apo-EV	apoptotic cell-derived EV
CAD	coronary artery disease
CD	cluster of differentiation
CRP-XL	cross-linked CRP peptides
DVT	deep vein thrombosis
EV	extracellular vesicle
FVL	factor V Leiden
HAEC	human arterial endothelial cell
HBMEC	human brain microvascular endothelial cell
HUVEC	human umbilical vein endothelial cell
ICAM-1	intracellular adhesion molecule-1
IL	interleukin
ISEV	International Society for Extracellular Vesicles
IVC	inferior Vena cava
LPS	lipopolysaccharide
MAPK	mitogen-activated protein kinase
MI	myocardial infarction
miR	microRNA
MP	microparticle
MV	microvesicle
NADPH	nicotinamide adenine dinucleotide phosphate
NO	nitric oxide
NTA	nanoparticle tracking analysis
PAI-1	plasminogen activator inhibitor-1
PAR	protease-activated receptor
PE	pulmonary embolism
PMA	phorbol myristate acetate
PS	phosphatidylserine
PSGL-1	P-selectin glycoprotein ligand-1 (CD162)
PCI	percutaneous coronary intervention
PTCA	percutaneous coronary angioplasty
RBC	red blood cell (erythrocyte)
ROS	reactive oxygen species
STEMI	ST-segment elevation myocardial infarction
TF	tissue factor
TFPI	tissue factor pathway inhibitor
THP-1	human monocytic leukemia cell line
TIM4	T-cell membrane 4
TNFα	tumor necrosis factor-alpha
TRAP6	thrombin receptor activator peptide-6
uPAR	urokinase type plasminogen activator receptor
VCAM-1	vascular cell adhesion molecule-1
VTE	venous thromboembolism
vWF	von Willebrand factor
